# Wrong Impressions

**Published:** 1898-01

**Authors:** 


### Editorial.

### WRONG IMPRESSIONS.

LANGUAGE is supposed to convey to other minds the thought of the individual using it, but, unfortunately, it does not always carry with it the motive which impels the utterance. This is apparent every day in life, presenting at times an amusing, at others a serious, side. It is probable that a large proportion of the antagonisms of the world follow misconceptions as to the spirit of the writer or speaker. This is the natural result of different states of mind.

This thought has received special prominence since the December issue of this journal. The writer of this took occasion, under the head of The Southern Dental Association, to quote the President of that body, in a few lines taken from the circular issued by himself. This was done not to antagonize the circular or the Southern, but simply to deprecate, in a few words, the *spirit* which seemed to the editor to be manifested,—that of a strong appeal to sectional feelings, which seemed to him out of place at this period.

In a pleasant letter received from Dr. Beadles, the President, in response to an explanatory letter from the writer, it was made evident that the editorial in question was regarded in a different light from that intended, and that the editor of this journal, possibly affected with mental strabismus, failed to discover the actuating motive of the circular. In view of this fact it is due Dr. Beadles that his reasons for its issue should be as prominently given as the editorial in question. The following quotation from his, specially relative to this, may answer. We would be pleased to give the letter entire did space permit.

Dr. Beadles writes, "Words seldom convey our real meaning. Your words made a different impression upon me from what you intended, so did mine upon you in my little circular. This circular was intended for the Southern men, and with it I was simply trying to arouse some interest through our *pride*, which is a strong factor with us. Not a thought of 'sectionalism,' in its objective sense, was in my mind. The truth is, the American and the Southern agreed to form a 'National Dental Association' rather than to *combine*. All of us recognized the wisdom of having one leading organization. The American had a perfect right to continue its existence, but as it did not, I see no obligation resting upon the Southern to go out of existence as a branch. You may not understand, but there is a peculiar necessity for the existence of this Association. It will not in any way interfere with the national organization, and will perform special work here in the South that could not possibly be done by the National. For this reason do we expressly desire to continue it. So my idea is that we should let things alone as they are for the present, say nothing nor do anything that will kindle hard feelings. If the plan does not work, some other may be adopted. I believe the *intention* of the Southern men was to *do right*, to do whatever was best for the profession at large, and then do what was best for our section."

The misconception of meaning in former editorials is apparent in this further quotation: "You say you have the kindest and best feelings towards the Southern, and I believe you, but heretofore all of your editorials upon this question have made a very different impression upon me. I was more than pleased that I was mistaken." This makes it imperative that the editor of this journal should plainly state what his ideas have been in regard to this matter of the union of the Southern with the American.

It is difficult to understand how any one could get the impression from any of the editor's writings that he was antagonistic to this union. For years he has been outspoken in regard to it, in conventions and wherever dentistry and things pertaining to its interest were considered. His hopes were centred at Old Point Comfort on this one thing. It was regarded by him as eminently proper that both the American and the Southern Dental Association should remain as organizations in their respective districts, but that a new body of an entirely different character from either, but of a higher order, should be formed. It was fully in accord with this idea that the Southern men decided not to make any change in name, but concluded to continue the Southern as a branch. This seemed to the writer to be a logical conclusion, and he was so much impressed with the fact that he retired from the presidential chair for the purpose of advocating a similar course for the American. This suggestion found but few supporters, and the American died and the Southern lived. In the opinion of the writer wisdom was on the side of the South.

Dr. Beadles expresses the hope that nothing will be done to "kindle hard feelings," and in this we are perfectly in accord, but while recognizing this, the editor of a representative dental journal has a higher duty to perform. He cannot stop to consider whether this or that one will be touched upon tender sensibilities, but must rise above mere personal prejudices, if any exist, and view things from a higher altitude. The man who cannot do this is unfit to conduct a journal. That all he writes should have the stamp of his individual character is a truism, but it does not follow that there need be illiberal treatment of subjects.

In conclusion, it is hoped that the Southern will be attended in February by those who can go from all sections of the South, and that it will then and there show to the North that the greatest mistake ever made was in destroying a thoroughly organized body, leaving in its place a dead memory.

We have faith in the good sense of the men in the ranks of dentistry, and look forward with hope to the establishing in the future of an organization worthy of its advanced culture. When that time comes we will have the Northern, Southern, and Western organizations working harmoniously with that higher body yet to be formed.

